# Endoscopic submucosal dissection combined surgery for the treatment of ectopic gastric mucosa and ectopic pancreas in muscularis propria and serosal layer of the stomach: A rare case report and review of the literature

**DOI:** 10.1097/MD.0000000000041297

**Published:** 2025-02-28

**Authors:** Yindi Chen, Yanbing Cao, Lingran Zhi, Rui Huang

**Affiliations:** a Department of Gastroenterology, Xi’an People’s Hospital (Xi’an Fourth Hospital), Xi’an, Shaanxi Province, China; b Department of General Surgery, Xi’an People’s Hospital (Xi’an Fourth Hospital), Xi’an, Shaanxi Province, China; c Department of Pathology, Xi’an People’s Hospital (Xi’an Fourth Hospital), Xi’an, Shaanxi Province, China.

**Keywords:** ectopic gastric mucosa, ectopic pancreas, endoscopic submucosal dissection, surgery

## Abstract

**Rationale:**

Ectopic gastric mucosa (EGM) and ectopic pancreas (EP) in the stomach is a rare congenital anomaly. No research on the coexistence of EGM and EP in the stomach has been found. However, several studies have shown canceration of EGM outside the stomach and EP. Active surgical treatment may be necessary. This paper introduces a new case of ectopic gastric mucosa and ectopic pancreas in muscularis propria and serosal layer of the stomach and which were ultimately removed by endoscopic submucosal dissection combined surgery.

**Patient concerns:**

The patient was a 25-year-old male. Upper gastrointestinal endoscopy showed a subepithelial lesion in gastric antrum.

**Diagnoses:**

Postoperative pathology confirmed a diagnosis of EGM and EP.

**Interventions:**

Enhanced computed tomography scan and endoscopic ultrasonography showed a submucosal tumor. Then endoscopic submucosal dissection was performed, during which a lesion was observed on the anterior wall of the stomach near the pylorus. This lesion invades the muscularis propria of the stomach and the large lesions near the pylorus may lead to pyloric obstruction. Considering these situations comprehensively, the patient ultimately underwent surgical resection of gastric lesion and partial gastrectomy.

**Outcomes:**

After 3 months of follow-up, the patient was recovering well and no recurrence of the lesion was found so far.

**Lessons:**

EGM along with EP in the stomach is a rare condition. Due to its rarity, there was a lack of sufficient data support for its treatment and prognosis. Due to the EGM and EP may become cancerous, clinical attention should be paid to it. In this study, we present a new case report of EGM along with EP in the stomach and review the existing literature to explore treatment options for it.

## 1. Introduction

Ectopic gastric mucosa (EGM) and ectopic pancreas (EP) is currently considered a rare congenital anomaly according to widely accepted theories. There were few studies on EGM in the stomach, to our knowledge, only 6 cases have been reported in the literature.^[1–6]^ And no research on the coexistence of EGM and EP in the stomach has been found. However, it has been noted that some patients with EGM or EP complaint nonspecific abdominal symptoms, even carcinogenesis cases have been shown. We have described here a case of EGM in muscularis propria and serosal layer of the stomach along with EP, resected by surgery combined endoscopic submucosal dissection (ESD) finally.

## 2. Case report

**Figure 1.**
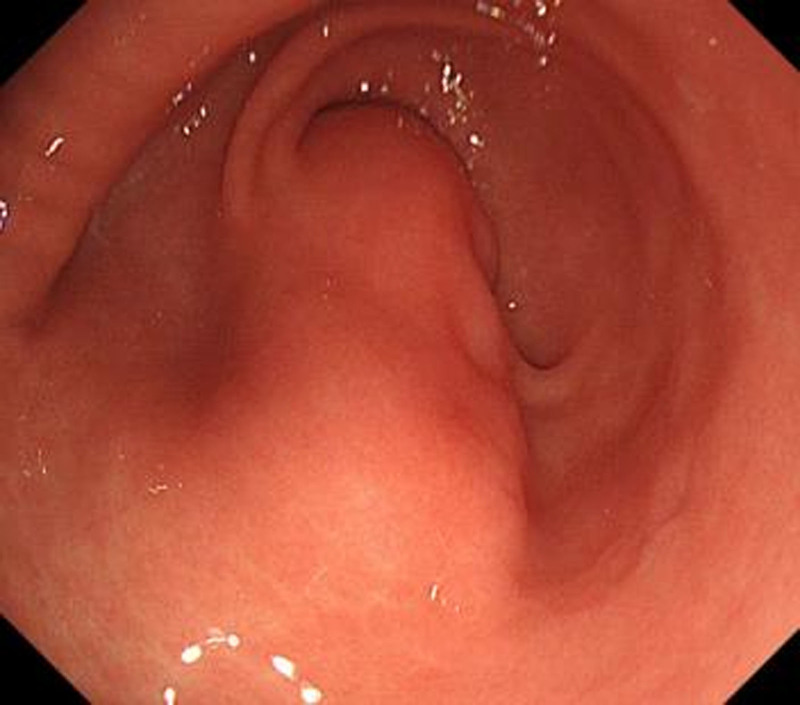
Upper gastrointestinal endoscopy showed a subepithelial lesion in gastric antrum.

A 25-year-old male visited in Xi’an People’s Hospital (Xian, China) for follow-up examination of gastric ulcer and he didn’t complain about any abdominal discomfort. The patient denied a family history of gastrointestinal tumors, the results of the physical examinations and laboratory tests showed no significant abnormalities. Upper gastrointestinal endoscopy showed a subepithelial lesion in gastric antrum (Fig. 1).

**Figure 2.**
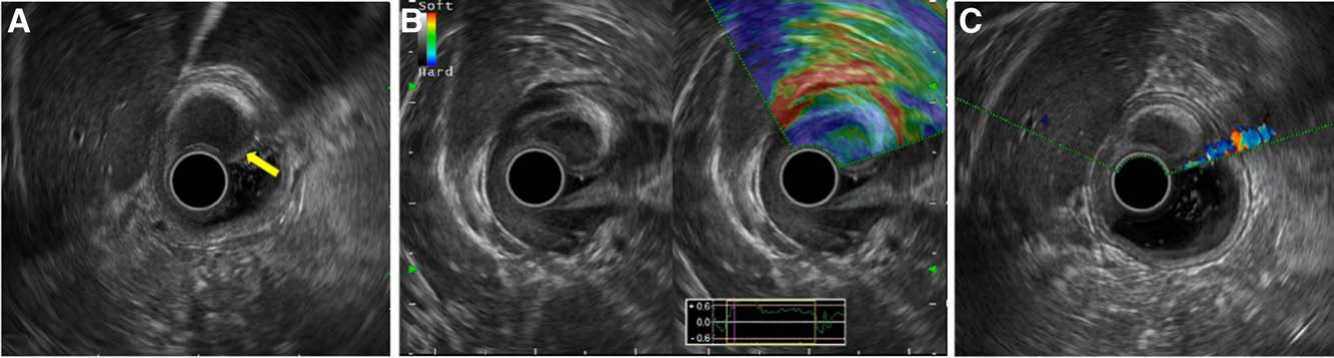
EUS showed a hypoechoic medium hard texture mass originating in the submucosal layer (A, B), originating in the submucosal layer, and CDFI showed no blood vessels inside the lesion (C). CDFI = color Doppler flow imaging, EUS = endoscopic ultrasonography.

**Figure 3.**
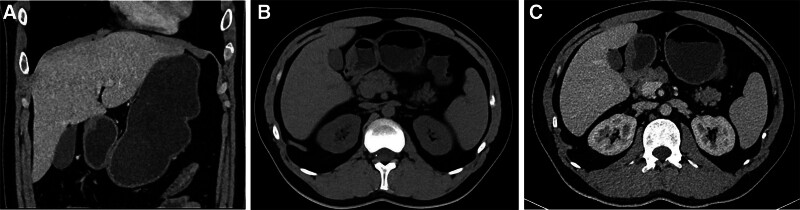
Abdominal enhanced computed tomography scan revealed an enhanced mass protruding into the stomach cavity.

To further clarify the diagnosis, endoscopic ultrasonography and enhanced computed tomography scan (CT) were performed. Endoscopic ultrasonography revealed a hypoechoic medium hard texture mass with an approximate 16.2 mm × 10 mm size, originating in the submucosal layer (Fig. 2A), and color Doppler flow imaging showed no blood vessels inside the lesion (Fig. 2B, C). Abdominal enhanced CT revealed an enhanced mass protruding into the stomach cavity (Fig. 3A–C). Based on the CT and endoscopy results, leiomyoma, gastrointestinal stromal tumor, and schwannoma were considered.

**Figure 4.**
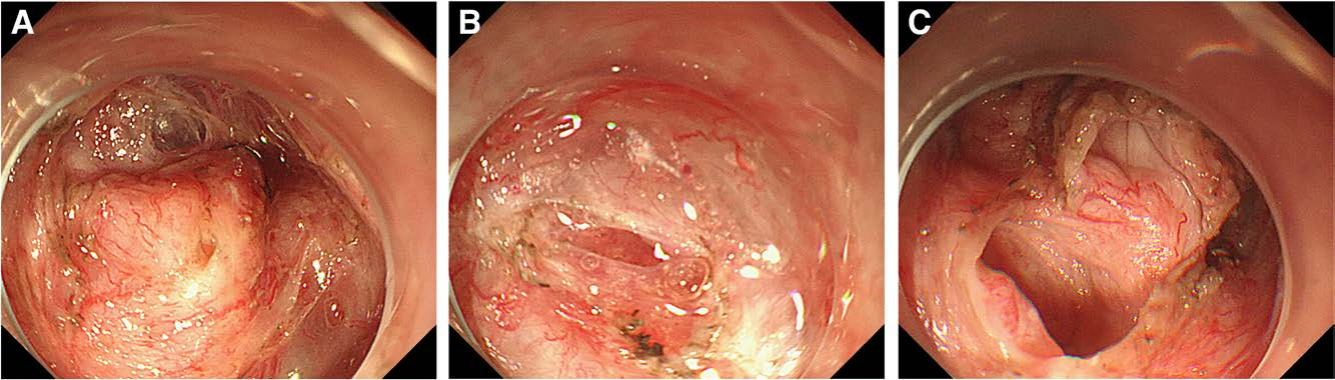
Process of endoscopic submucosal dissection (ESD). (A) An peristaltic, irregular with unclear boundaries mass under the endoscope. (B) A small defect with transparent mucus on the surface of the mass and light yellow flocculent material flowing out. (C) A digestive tract lumen with blind ends on both sides and smooth inner wall surfaces after the lesion was cut. ESD = endoscopic submucosal dissection.

**Figure 5.**
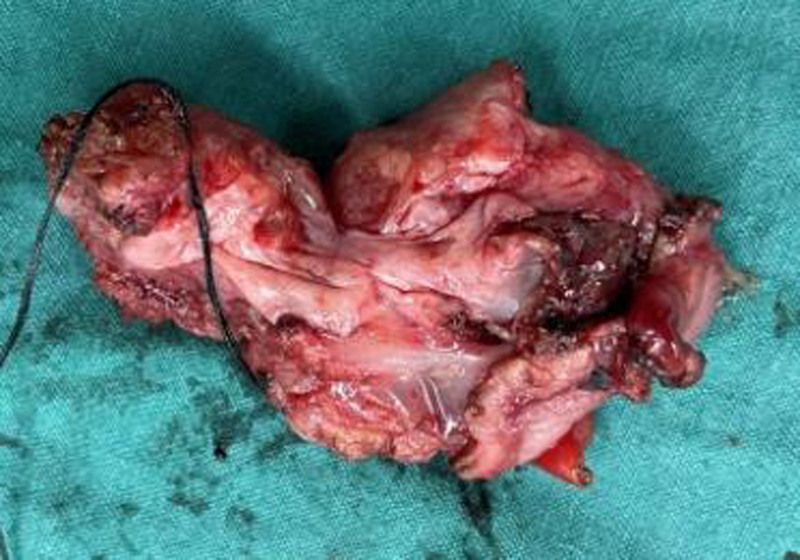
Surgical specimen. A 2 cm × 3 cm sized hard mass was observed on the anterior wall of the stomach near the pylorus during the surgery.

Considering the large size of the lesion, the postoperative specimen may not be completely removed from the digestive tract lumen after ESD. Therefore, after obtaining the patient’s written informed consent, we ultimately decided to combined endoscopic and laparoscopic in operating room to remove the lesion and ensure its integrity. While creating a submucosal tunnel toward the tumor under the endoscope, an peristaltic, irregular with unclear boundaries mass was seen in the submucosa. During ESD, vascular bleeding was observed on the surface of the lesion. We performed electrocoagulation and discovered a small defect during the process, with transparent mucus and light yellow flocculent material flowing out (Fig. 4A, B). Gradually separating the lesion along the contour of the lesion, no clear boundaries between the lesion and the intrinsic muscle layer were found. The surface of the lesion was cut during separating the lesion along the intrinsic muscle layer, and the interior was exposed, which was similar to a digestive tract lumen with blind ends on both sides and smooth inner wall surfaces (Fig. 4C). We suspected that the tumor may be a rare alimentary tract duplication and the large lesion can easily lead to pyloric obstruction. Then laparoscopic exploration and gastric lesion resection was performed. During the surgery, a 2 cm × 3 cm sized hard mass was observed on the anterior wall of the stomach near the pylorus (Fig. 5), which was beyond the serosal layer and adhering to the transverse colon and liver.

**Figure 6.**
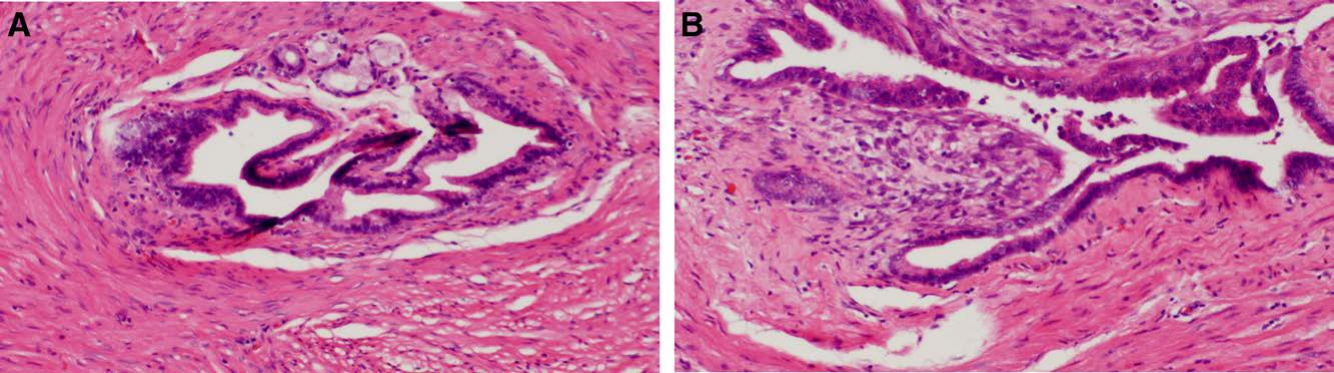
Histopathological appearance. Surgical specimen was confirmed ectopic gastric mucosa and ectopic pancreas that consists only of ducts. (A) Magnification, ×100; (B) magnification, ×200, H&E staining.

Histological examination of the tumor confirmed gastric mucosal ectopia and EP that consists only of ducts (Fig. 6A, B). After 3 months of follow-up, the patient was recovering well, with no recurrence of the lesion observed.

## 3. Discussion

EGM refers to the ectopic transfer of normal gastric mucosal tissue to other organs, most commonly occurring in the esophagus, duodenal bulb, and Michael’s diverticulum, it has been also identified in the nasopharynx,^[7]^ gallbladder,^[8]^ and in the rectum.^[9]^ However, there was a rare occurrence of EGM within the stomach itself. The pathogenesis mainly includes embryo remnants and abnormal proliferation associated with inflammation, the former theory is the most widely accepted.^[1]^ In this report, a 25-year-old patient had a history of gastric ulcers. The “abnormal proliferation associated with inflammation” theory may be apply to this patient, and EGM of the stomach could have been the result of chronic inflammation. EGM has no specific symptoms and is often discovered during endoscopic examination or surgery. Symptoms often occur due to peptic ulcers caused acid secreting,^[10]^ and some lesions can lead to gastrointestinal obstruction,^[11]^ spontaneous fistula,^[12]^ and intussusception.^[13]^ There were several studies reporting the carcinogenesis EGM.^[14–18]^ EGM is difficult to distinguish from neuroendocrine tumor, gastric stromal tumor and EP under gastroscopy, a histopathological diagnosis is required. Gastric foveolar epithelium, the chief cells and gastric fundic gland cells in its depth are identified histologically, can be diagnosed as EGM.

EP refers to isolated pancreatic tissue that grows outside the pancreas itself and has no anatomical or vascular connection with normal pancreatic tissue.^[19]^ The incidence rate of EP is 0.5% to 3.7%,^[20]^ and it is more commonly occurring in the digestive tract, especially in the gastric antrum.^[19]^ It is also visible in the other areas such as jejunum,^[21]^ gallbladder,^[22]^ and Meckel’s diverticulum.^[23]^ The pathogenesis of EP remains elusive to date, and most opinions believe that the congenital embryonic developmental abnormalities lead to the development of EP.^[20]^ EP is mostly submucosal lesions, and it is difficult to obtain valuable diagnostic specimens through gastroscopy biopsy that resulting in a low positive rate. The gold standard for the diagnosis of EP depends on the histopathology after surgical resection. The Gaspar-Fuentes classification^[24]^ is used to classify EP into 4 types. In Type I, in which all components of the ducts, acini, and islets are observed. Type II consists only of ducts. Type III consists only of acini, Type IV consists only of islets. The final pathology of our case belongs to the Type II EP. Most cases of EP are asymptomatic and not easily detected. Researches have showed that EP may lead to acute abdominal symptoms,^[23]^ bleeding^[25]^ and malignant transformation.^[26]^ Armstrong et al^[27]^ found that a maximum diameter of EP tissue >1.5 cm was associated with signs and symptoms of it. This may be closely related to the secretion of proteolytic enzymes leading to the damage of the mucosal barrier of the digestive tract or chronic inflammation and ulcers of the digestive tract caused by mucosal atrophy in the corresponding area due to EP compression. Therefore, EP should be closely followed up and actively treated.

To our knowledge, this is the first case of EGM in muscularis propria and serosal layer of the stomach along with EP and undergoing gastroscopy combined with laparoscopic resection. Of course, there is some limitations in this study, including the lack of long-term follow-up, so that it is currently impossible to evaluate the long-term effectiveness of treatment and the potential risk of recurrence.

## 4. Conclusion

This is a rare case of gastric mucosal ectopia in muscularis propria and serosal layer of the stomach along with EP. Due to the EGM and EP may become cancerous, misdiagnosis should be avoided as much as possible and active treatment such as endoscopic or surgical resection may be necessary.

## Author contributions

**Conceptualization:** Yindi Chen, Rui Huang.

**Data curation:** Yindi Chen, Yanbing Cao, Lingran Zhi, Rui Huang.

**Writing – original draft:** Yindi Chen.

**Writing – review & editing:** Rui Huang.
